# Antigen-specific chemokine profiles as biomarkers for detecting *Mycobacterium tuberculosis* infection

**DOI:** 10.3389/fimmu.2024.1359555

**Published:** 2024-03-06

**Authors:** Weicong Ren, Zichun Ma, Qiang Li, Rongmei Liu, Liping Ma, Cong Yao, Yuanyuan Shang, Xuxia Zhang, Mengqiu Gao, Shanshan Li, Yu Pang

**Affiliations:** ^1^ Department of Bacteriology and Immunology, Beijing Chest Hospital, Capital Medical University/Beijing Tuberculosis and Thoracic Tumor Research Institute, Beijing, China; ^2^ Department of Tuberculosis, Beijing Chest Hospital, Capital Medical University/Beijing Tuberculosis and Thoracic Tumor Research Institute, Beijing, China

**Keywords:** tuberculosis, latent tuberculosis, chemokine, biomarker, IGRA

## Abstract

**Background:**

Latent tuberculosis (TB) infection can progress to active TB, which perpetuates community transmission that undermines global TB control efforts. Clinically, interferon-γ release assays (IGRAs) are commonly used for active TB case detection. However, low IGRA sensitivity rates lead to false-negative results for a high proportion of active TB cases, thus highlighting IGRA ineffectiveness in differentiating MTB-infected individuals from healthy individuals.

**Methods:**

Participants enrolled at Beijing Chest Hospital from May 2020-April 2022 were assigned to healthy control (HC), LTBI, IGRA-positive TB, and IGRA-negative TB groups. Screening cohort MTB antigen-specific blood plasma chemokine concentrations were measured using Luminex xMAP assays then were verified via testing of validation cohort samples.

**Results:**

A total of 302 individuals meeting study inclusion criteria were assigned to screening and validation cohorts. Testing revealed significant differences in blood plasma levels of CXCL9, CXCL10, CXCL16, CXCL21, CCL1, CCL19, CCL27, TNF-α, and IL-4 between IGRA-negative TB and HC groups. Levels of CXCL9, CXCL10, IL-2, and CCL8 biomarkers were predictive for active TB, as reflected by AUC values of ≥0.9. CXCL9-based enzyme-linked immunosorbent assay sensitivity and specificity rates were 95.9% (95%CI: 91.7-98.3) and 100.0% (92.7-100.0), respectively. Statistically similar AUC values were obtained for CXCL9 and CXCL9-CXCL10 assays, thus demonstrating that combined analysis of CXCL10 and CXCL9 levels did not improve active TB diagnostic performance.

**Conclusion:**

The MTB antigen stimulation-based CXCL9 assay may compensate for low IGRA diagnostic accuracy when used to diagnose IGRA-negative active TB cases and thus is an accurate and sensitive alternative to IGRAs for detecting MTB infection.

## Introduction

Tuberculosis (TB), an infectious disease caused by *Mycobacterium tuberculosis* (MTB) complex, remains a leading killer worldwide (1, 2). Globally, an estimated 10.6 million new TB cases and approximately 1.6 million TB-related deaths were reported in 2021 (2). It is worth mentioning that one-third of the world’s population is infected with MTB, of which only 5%–10% of infected individuals develop active TB disease over their lifetimes (3, 4). However, most MTB-infected individuals harbor viable, quiescent tubercle bacilli without exhibiting clinical symptoms of active TB, a state known as latent tuberculosis infection (LTBI) (5). The large pool of individuals with LTBI constitutes a major source of subsequent active TB cases, which perpetuates community TB transmission. This issue impedes efforts toward achieving the END TB Strategy goal of eliminating TB by 2035 (5).

Detection and effective management of TB infection are considered core interventions for achieving global TB elimination (6). Conventionally, tuberculin skin testing (TST) is widely used for TB infection screening, but lacks specificity that may lead to false-positive test results for individuals with histories of bacillus Calmette-Guérin (BCG) vaccination and/or nontuberculous mycobacteria (NTM) exposure (7, 8). In addition, the TST frequently yields false negative results when used to test immunocompromised patients (9). Recently, new tests that detect blood cytokine levels have been developed to identify TB-infected individuals (10). Tests that measure interferon-γ (IFN-γ) secretion by peripheral blood T-lymphocytes in response to stimulation with mycobacterial antigens, IFN-γ release assays (IGRAs). As compared to the TST, IGRAs can provide greater TB detection specificity (10) and greater sensitivity when used to test children and elderly individuals (11, 12). Thus, IGRAs are currently viewed by healthcare providers as preferred assays for diagnosing MTB-infected individuals with symptoms suggestive of active TB, such that negative IGRA results are used to rule out TB in clinical practice. However, IGRAs generate false-negative results for a substantial proportion of MTB-infected individuals as a reflection of unacceptably low IGRA sensitivity in differentiating between MTB-infected and healthy individuals (13, 14). Meanwhile, results of several other studies have shown that old age, overwhelming active TB infection, HIV infection, and low lymphocyte count are factors that increase an individual’s risk of receiving false-negative IGRA results (15), such that negative IGRA results cannot be used to rule out active TB status for individuals with these risk factors. More importantly, MTB-infected patients with false-negative IGRA results who are started on immunotherapy without first receiving anti-TB treatment are theoretically at increased risk of developing active TB. Therefore, new assays based on detection of MTB antigen-specific host biomarkers are needed in order to compensate for low IGRA sensitivity.

Chemokines, which are proinflammatory cytokines that are released at sites of infection, inflammation, and injury, orchestrate migrations of specific types of leukocytes to infected tissues (16). During initial pulmonary MTB infection and TB progression stages, induction of inflammatory chemokines results in recruitment of newly activated effector T cells that ultimately perpetuate an inflammatory state that can effectively control bacterial replication in the lungs (17). In fact, results of recent studies have pinpointed the CXCL10 chemokine as a potentially useful TB-diagnostic biomarker (18), while recently reported results of a study conducted in India demonstrated that higher chemokine levels were associated with greater TB disease severity and greater bacterial burden (19). However, no reported studies have assessed chemokine levels of individuals with false-negative IGRA results, warranting further research to discover and validate additional candidate biomarkers for this population. In this study, we first identified chemokines with potential utility as MTB infection-specific biomarkers through comparative analysis of MTB antigen-specific chemokine profiles obtained for blood samples of a screening cohort that included healthy subjects and subjects with LTBI or active TB. Potentially useful MTB infection-associated chemokine biomarkers were further evaluated for specificity and sensitivity via testing of blood samples obtained from a larger validation cohort.

## Methods

### Study population

**Figure 1:**
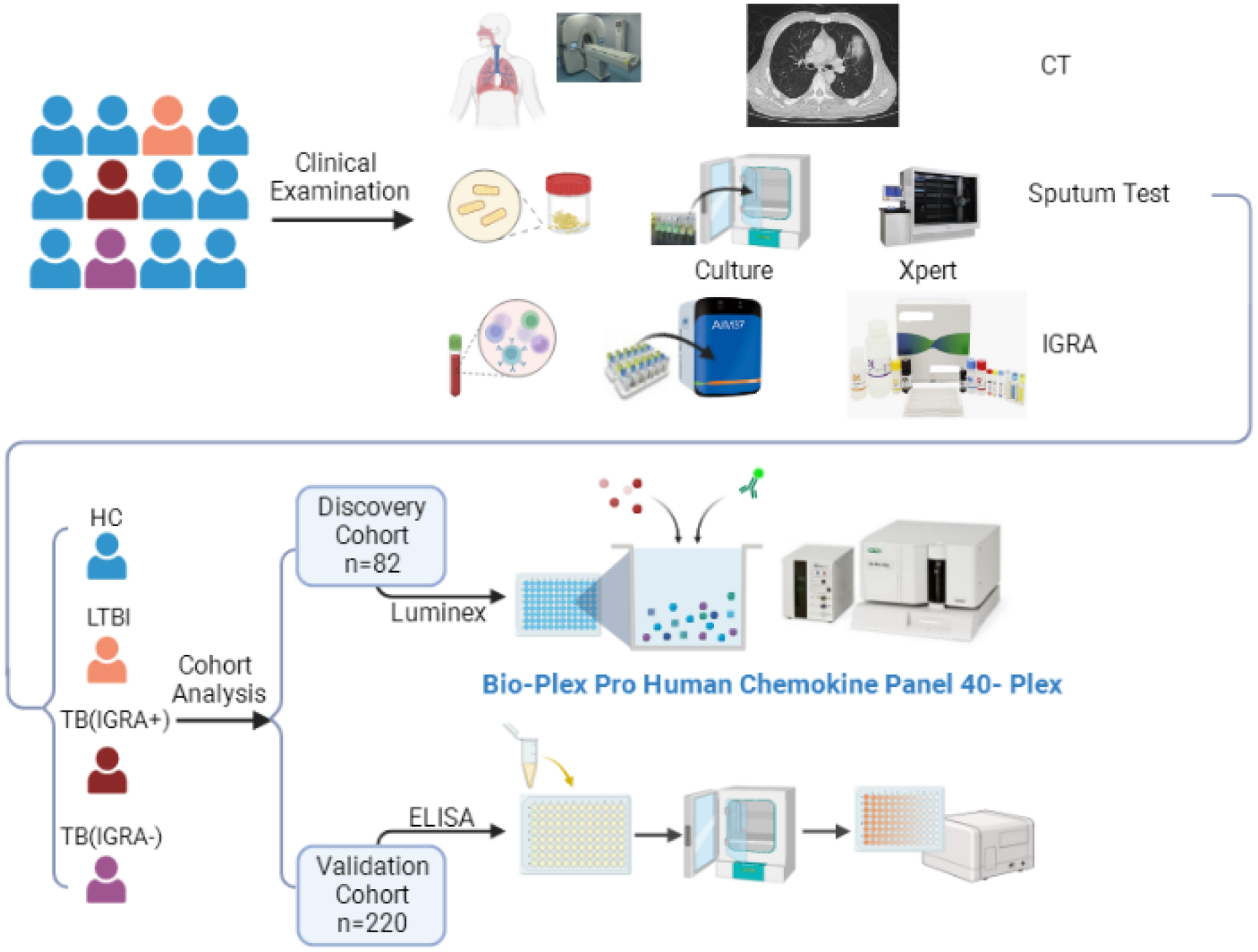
The workflow of this study. CT, Computed Tomography; IGRA, Interferon-γ Release Assays; TB, Tuberculosis; LTBI, Latent Tuberculosis Infection; ELISA, Enzyme Linked Immunosorbent Assay.

Participants were consecutively enrolled in this study who had served as subjects of a previous cohort study conducted at Beijing Chest Hospital between May 2020 and April 2022. All participants enrolled in the current study exhibited symptoms suggestive of active TB. Sputum and blood specimens were collected for smear microscopy, mycobacterial culture (Becton Dickinson, Sunnyvale USA), GeneXpert MTB/RIF testing (Cepheid, Sunnyvale USA), and IFN-γ release assays (Leide, Guangzhou China), as shown in **Figure 1**. Only active TB patients with positive test results demonstrating the presence of tubercle bacilli in sputa were included in the study. Participants were then assigned to IGRA-positive and IGRA-negative groups based on IGRA test results. Meanwhile, latent TB infection (LTBI) and healthy control (HC) groups were assembled that included volunteers with normal chest radiographic results and a lack of clinical symptoms suggestive of active TB; IGRAs were conducted for all members of these groups. Next, 20 or 21 individuals from each of the four abovementioned groups were randomly assigned to the screening cohort, while all other individuals were assigned to the external validation cohort. The study protocol was approved by the Ethics Committee of Beijing Chest Hospital, a hospital affiliated with Capital Medical University (approval number: YJS-2019-016). Informed consent was obtained from each participant prior to enrollment in the study.

### IFN-γ release assays

Peripheral whole blood was collected from all study participants using heparin anticoagulant blood collection tubes (Becton, Dickinson and Company, USA) then each blood sample was directly transferred to three tubes (0.6 ml of blood/tube) that included the negative control (N tube), TB antigen (T tube), and positive control (P tube), as recommended by the manufacturer (Leide, Guangzhou China). Tubes were incubated at 37°C for 20 ± 2 h in an incubator containing 5% CO_2_. Tubes were next centrifuged at 1000 × *g* for 5 min. Thereafter, ∼150 μl of blood plasma was transferred to a new tube for further analysis then the IFN-γ concentration of a 50-μl volume of plasma was measured using a commercial sandwich enzyme-linked immunosorbent assay (ELISA) kit (Leide, Guangzhou China). The remaining plasma volume was stored at -80°C then tested for levels of numerous chemokines using a multiplex chemokine assay kit and for CXCL10 and IFN-γ-inducible protein-10 (IP-10) levels using ELISA kits. Values of MTB-specific antigen-stimulated cytokines were determined by subtracting negative control (N) values from TB antigen (T) values then calculated values were interpreted as positive or negative results based on a selected cut-off value (≥20 pg/ml), as indicated in the manufacturer’s instructions (Leide, Guangzhou China).

### Multiplex chemokine assay

Chemokine concentrations of plasma samples were measured using the Bio-Plex Pro Human Chemokine Panel 40-plex kit (Bio-Rad, CA, USA), with Wayen Biotechnologies (Shanghai, China) performing all chemokine concentration determinations according to the manufacturer’s instructions. Briefly, each plasma sample was added to a 96-well plate well containing embedded microbeads. Next, 96-well plates containing samples were incubated for 1 h then detection antibody was added to wells followed by incubation of plates for an additional 30 min. Thereafter, wells were washed then streptavidin-phycoerythrin (PE) was added to wells. Next, 96-well plates were incubated for 10 min then fluorescence intensities of samples and standards in wells were measured using a Bio-Plex 200 System (Luminex Corporation, Austin, TX, USA). In the final step, fluorescence results were calculated and normalized then output files were generated using Microsoft Excel.

### ELISA

Plasma CXCL9 and IP-10 levels were measured using commercial sandwich ELISAs (QuantiCyto^®^ Human MIG/CXCL9 and QuantiCyto^®^ Human IP-10/CXCL10 ELISA kits, NeoBioscience, Guangzhou, China) according to the manufacturer’s instructions.

### Statistical analysis

All statistical analyses were performed using SPSS version 20.0 software (IBM Corp., Armonk, NY) in combination with GraphPad Prism 8.0 software (GraphPad). Continuous variables were expressed as median (range) values and categorical variables were expressed as percentages (%). Significant differences between groups were evaluated for statistical significance using the Kruskal-Wallis test coupled with Dunn’s correction for multiple comparisons. The diagnostic performance of each chemokine-based assay was evaluated based on receiving operating characteristic (ROC) curves then assay sensitivity and specificity rates were estimated using Youden’s index. The proportion of correctly diagnosed patients was calculated based on its proportionality to the total area-under-the-curve (AUC) value. Differences between groups were declared significant for results with two-sided *P* values of <0.05.

## Results

### Classification of screening groups

In this study, a total of 302 individuals meeting study inclusion criteria (51.32% male, mean age of 42 years) were assigned to two cohorts: a screening cohort and an external validation cohort. Demographic and clinical characteristics of study participants are provided in **Table 1**. The screening cohort of 82 subjects included 20 HC subjects, 20 LTBI subjects, 21 IGRA-positive TB patients, and 21 IGRA-negative TB patients. The remaining 220 subjects were assigned to the external validation cohort, including 49 HC subjects, 32 LTBI subjects, 103 IGRA-positive TB patients, and 36 IGRA-negative TB patients.

**Table 1:** Clinical and demographic characteristics of participants.

Patient characteristics	HC (n=69)	LTBI (n=52)	TB (IGRA+) (n=124)	TB (IGRA-) (n=57)	Total (n=302)
**Age in years, median (IQR)**	35 (25-54)	35 (26-53)	42 (13-93)	55 (31-63)	42(23-64)
Sex, n (%)					
Male	27 (39.13%)	23 (44.44%)	67 (54.03%)	38(66.7%)	155 (51.32%)
Female	42 (60.87%)	29 (55.56%)	57 (45.97%)	19(33.3%)	147 (48.68%)
Clinical examination					
CT Positive	–	–	53	32	85
Culture Positive	–	–	51	29	90
Xpert positive	–	–	93	33	126
IGRA Positive	–	–	96	0	96
Complication					
Diabetes mellitus	–	–	17	7	24
Liver Disease	–	–	29	17	46
Kidney Disease	–	–	2	1	3
Hypertension/CHD	–	–	8	3	11
Cerebral infarction	–	–	5	2	7

HC, Healthy Control; LTBI, Latent TB Infection; TB, Tuberculosis; CT, Computed Tomography; IGRA, IFN-γ release assays.

### Levels of CCL chemokines, CXCL chemokines, and other cytokines for four groups of subjects

**Figure 2:**
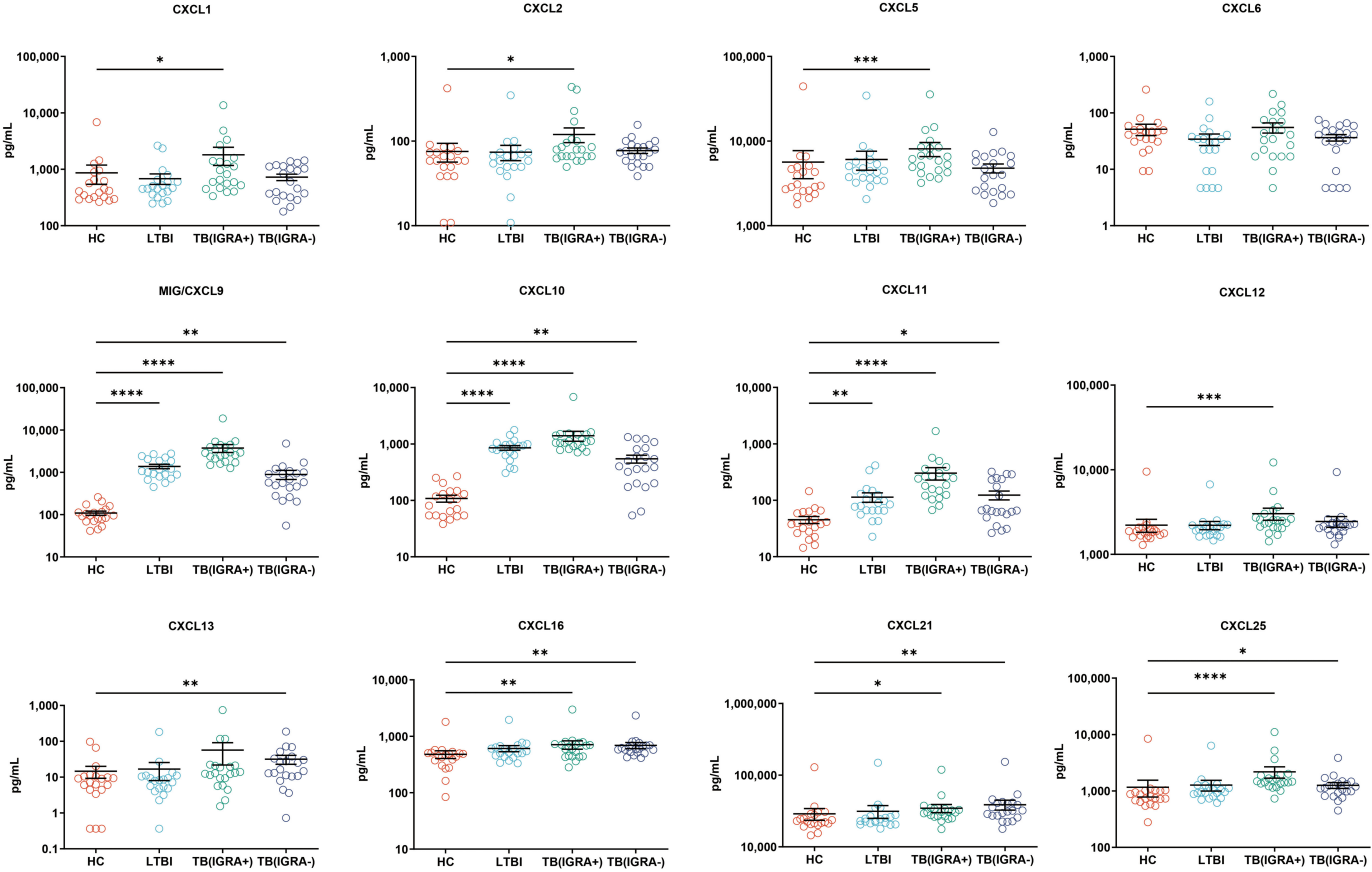
Median levels of CXCL chemokines (pg/mL) in blood cell supernatants of HC, LTBI, IGRA-positive TB cases, and IGRA-negative TB cases. Significant differences in chemokine levels between groups were detected using the Kruskal-Wallis test coupled with Dunn’s correction for multiple comparisons and expressed as *(*P* < 0.05), **(*P* < 0.01), ***(*P* < 0.001), ****(*P* < 0.0001).

**Figure 3:**
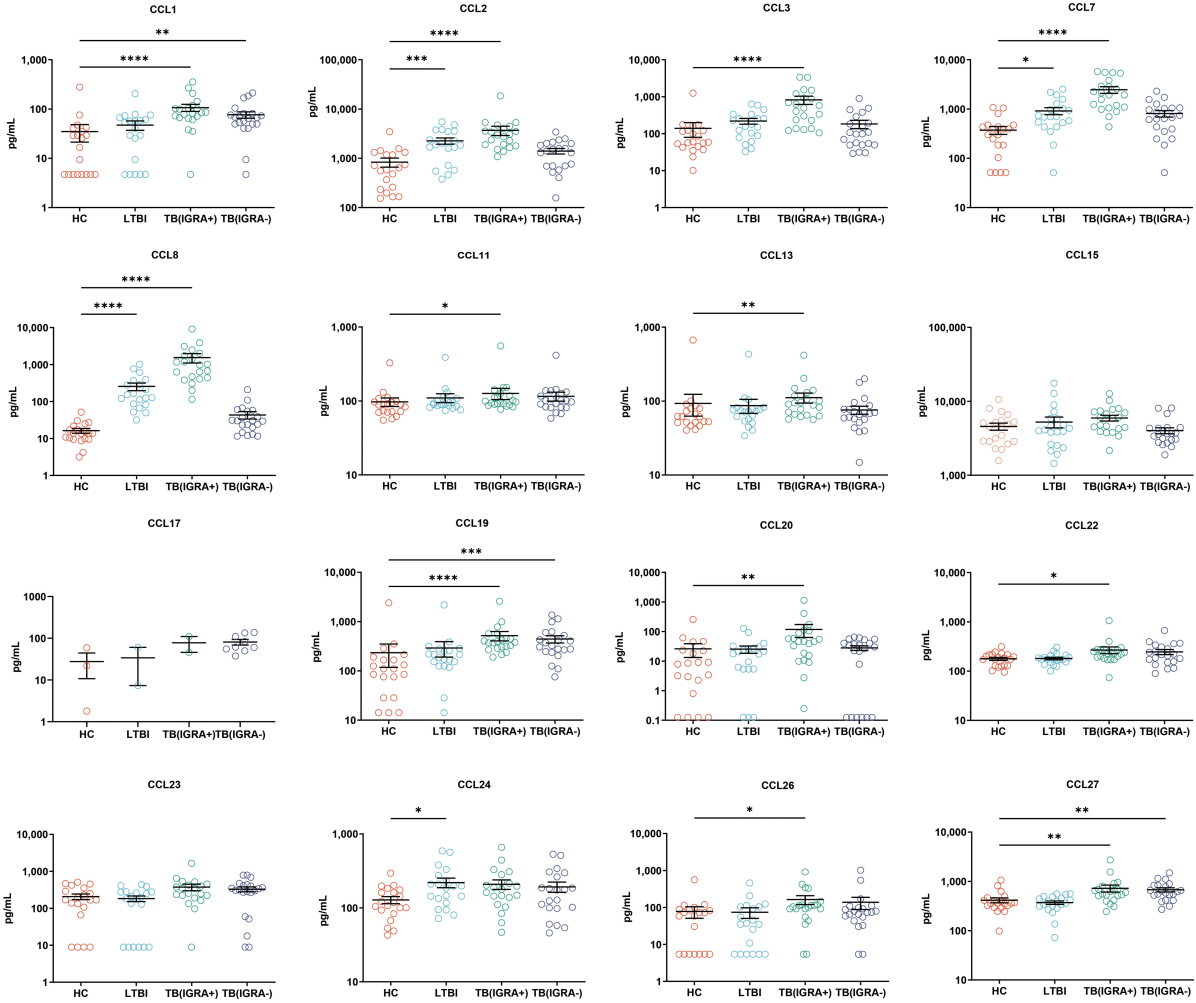
Median levels of CCL chemokines (pg/mL) in supernatants of blood cells of HC, LTBI, IGRA-positive TB and IGRA-negative TB cases. Significant differences in chemokine levels between groups were detected using the Kruskal-Wallis test coupled with Dunn’s correction for multiple comparisons and expressed as: *(*P*<0.05), **(*P* < 0.01), ***(*P* < 0.001), ****(*P* < 0.0001).

**Figure 4:**
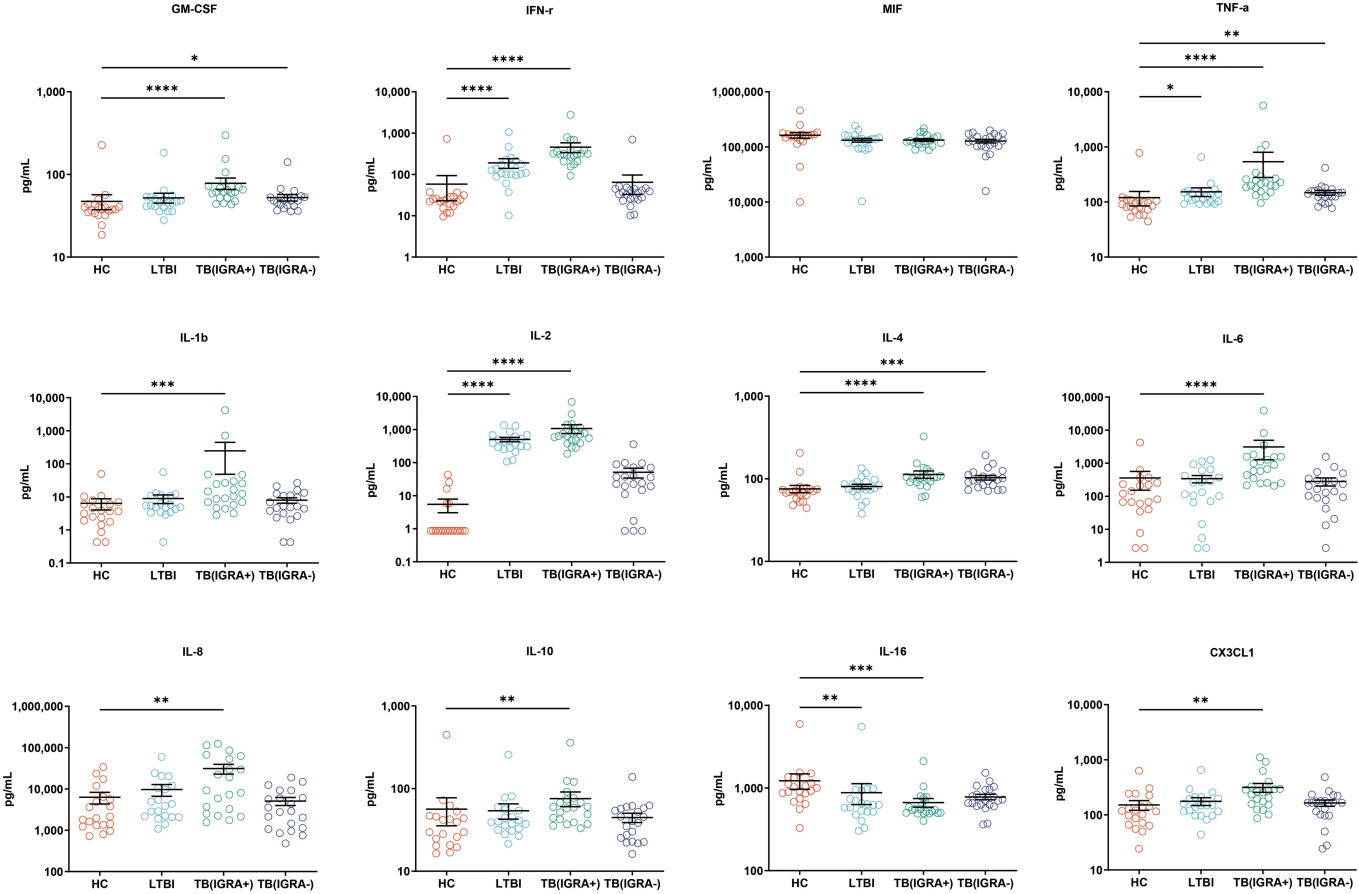
Median levels of other cytokines (pg/mL) in supernatants of blood cells HC, LTBI, IGRA-positive TB and IGRA-negative TB cases. Significant differences between groups were detected using the Kruskal-Wallis test coupled with Dunn’s correction for multiple comparisons and expressed as: *(*P* < 0.05), **(*P <* 0.01), ***(*P <* 0.001), ****(*P <* 0.0001).

In this study, a Luminex High Performance Assay kit was used to detect levels of 40 cytokines in supernatants of MTB antigen-stimulated blood cells. Thereafter, results obtained for the HC group and the three groups of MTB-infected subjects were compared based on intergroup differences in cytokine levels, with results shown in **Figures 2**-**4**. The results revealed that IGRA-positive TB group levels exceeded corresponding HC group levels obtained for multiple chemokines and cytokines, including CXCL6, CXCL9, CXCL10, CXCL11, CXCL12, CXCL16, CXCL25, CCL1, CCL2, CCL3, CCL7, CCL8, CCL19, CCL20, CCL27, GM-CSF, IFN-γ, TNF-α, IL-1b, IL-2, IL-4, IL-6, IL-8, IL-10, IL-16, and CX3CL1 (*P <* 0.01). Significant differences in levels of CXCL9, CXCL10, CXCL16, CXCL21, CCL1, CCL19, CCL27, TNF-α, and IL-4 between the IGRA-negative TB group and the HC group were also observed (*P <* 0.01). Moreover, LTBI group, IGRA-positive TB group, and IGRA-negative TB group levels of CXCL9 and CXCL10 were significantly different from corresponding HC group levels (*P <* 0.01). Median plasma cytokine levels (expressed as pg/ml) and their corresponding standard error-of-the-mean (SEM) values for the four abovementioned groups are presented in **Figures 2**–**4**.

### Performance of biomarkers in predicting MTB infection

**Figure 5:**
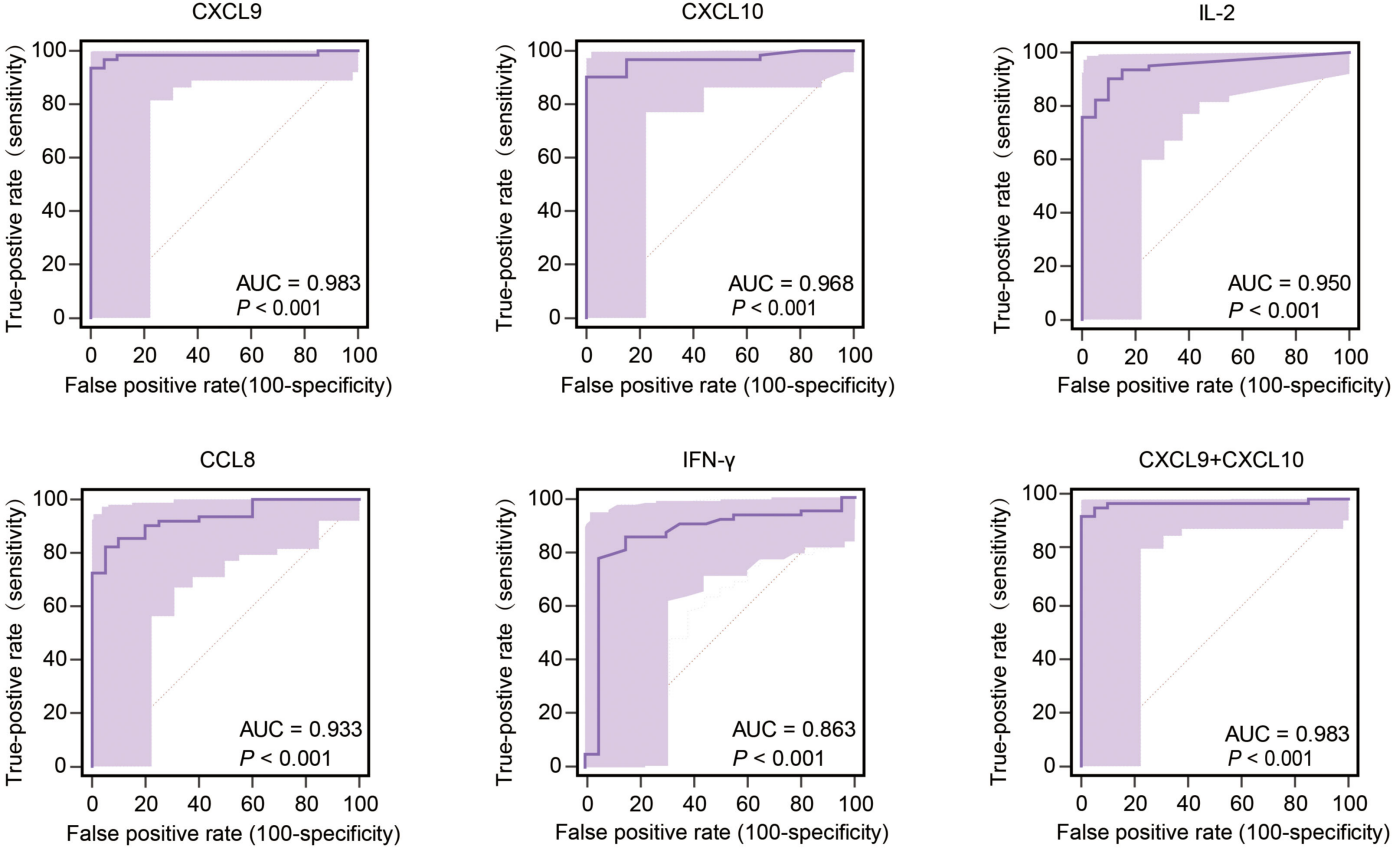
Performance of the most effective biomarkers for predicting TB infection. AUC, area under the curve.

Performance characteristics of biomarkers for predicting TB infection status were evaluated for sensitivity and specificity by constructing ROC curves that were used to calculate area-under-the-curve (AUC) values with 95% confidence intervals (CI). Intergroup comparisons of AUC values, which served as indicators of biomarker performance in predicting MTB infection status, ultimately revealed that higher AUC values corresponded to better performance in predicting MTB infection status. Analysis of AUC values obtained for the 40 tested biomarkers revealed highest AUC values for CXCL9, CXCL10, IL-2, and CCL8 (listed in decreasing order), all of which were significantly higher than the AUC value obtained for IFN-γ (**Figure 5**). Furthermore, AUC values obtained for CXCL9, CXCL10, IL-2, and CCL8 biomarkers were ≥0.9, thus highlighting their promise as biomarkers for use in predicting MTB infection status. Of note, CXCL9 and CXCL10 biomarkers performed best, as reflected by AUC values of 0.983 (95% CI: 0.926-0.999) and 0.968 (95% CI: 0.904-0.994), respectively, that exceeded AUC values of all other biomarkers. The CXCL9 specificity rate was 100% (95% CI: 83.2-100.0) and was the same as the CXCL10 sensitivity rate, while the CXCL9 biomarker sensitivity rate of 93.6% (95% CI: 84.3-98.2) was greater than the CXCL10 sensitivity rate of 90.3% (95%CI: 80.1-96.4). The IL-2 sensitivity rate was 90.3% (95% CI: 80.1-96.4) and the IL-2 specificity rate was 90.0% (95% CI: 68.3-98.8). The CXCL9 cut-off value for predicting MTB infection was 258.78 pg/ml, while the corresponding CXCL10 cut-off value was 267.53 pg/ml. Notably, the AUC value obtained when CXCL9 and CXCL10 biomarkers were analyzed together did not statistically differ from the AUC value obtained for the CXCL9 biomarker alone (**Figure 5**), thus indicating that both assays predicted MTB infection equally well. Therefore, no improvement in CXCL9 biomarker performance in detecting MTB infection was observed after CXCL10 biomarker data were included in the analysis.

### Assessments of biomarker levels and diagnostic performance for the validation cohort

**Figure 6:**
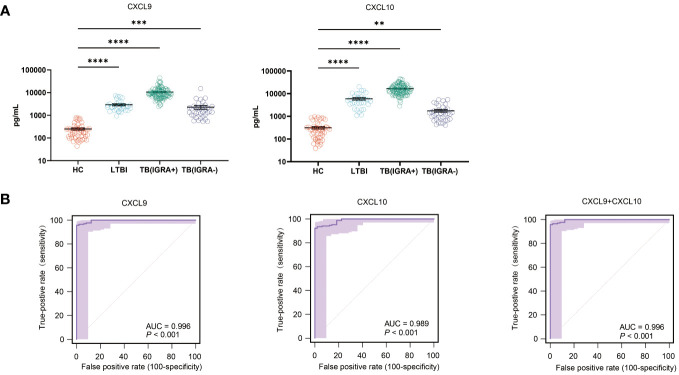
Intergroup differences in CXCL9 and CXCL10 levels and TB diagnostic performance. **(A)** Plasma CXCL9 and CXCL10 level differences between IGRA-positive TB, IGRA-negative TB, latent TB infection (LTBI), and healthy control (HC) groups, as determined using ELISAs [*(*P* < 0.05), **(*P <* 0.01), ***(*P <* 0.001), ****(*P <* 0.0001)]. **(B)** Differences in receiving operating characteristic (ROC) curve-based results between LTBI, IGRA-positive TB, IGRA-negative TB, and HC groups as based on area-under-the-curve (AUC) values.

Intergroup differences in levels of CXCL9 and CXCL10 were verified via ELISA-based testing of validation cohort samples. Differences of validation cohort samples were as same as the results of tests before, as shown in **Figure 6A**. The CXCL9 sensitivity rate was 95.9% (95% CI: 91.7-98.3), its specificity rate was 100.0% (95% CI: 92.7- 100.0), and its AUC value was 0.996 (cut-off: 793.71 pg/ml). Meanwhile, the CXCL10 assay sensitivity rate was 92.4% (95% CI: 87.4-95.9), its specificity rate was 100.0% (95% CI: 92.7- 100.0), and its AUC value was 0.924 (cut-off: 951.28 pg/ml) (**Figure 6B**). When CXCL9 and CXCL10 assays were combined into a single assay, the TB detection accuracy of the resulting combined assay was equal to that of the CXCL9 assay, thus indicating that inclusion of CXCL10 levels in the analysis did not increase the diagnostic accuracy of the CXCL9 assay (**Figure 6B**).

### The relationship between biomarker levels and IFN-γ level

**Figure 7:**
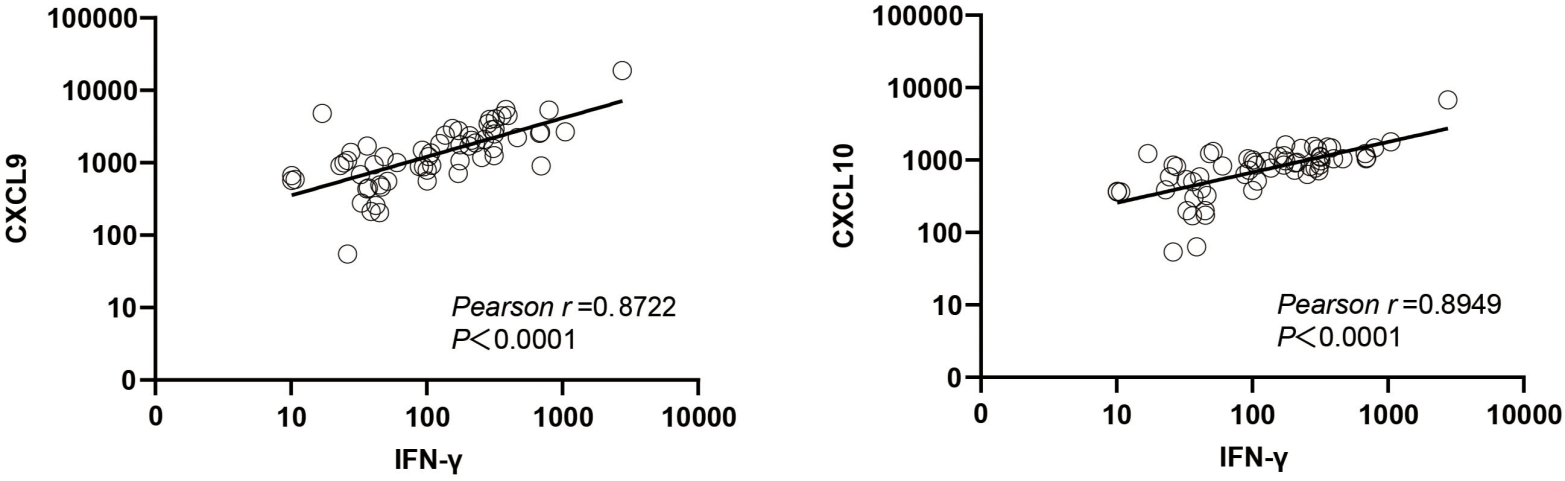
Correlation between IFN-γ and two novel biomarkers.

Chemokines CXCL9 (an interferon-induced monokine) and CXCL10 (IP-10) are produced by various types of host cells in response to IFN-γ stimulation (20). Here the plasma CXCL9 concentration was found to be positively correlated with the plasma IFN-γ concentration (r = 0.8722, *P <* 0.0001), as was the plasma CXCL10 concentration (r = 0.8949, *P <* 0.0001) (**Figure 7**).

## Discussion

Accurate and timely diagnosis of TB infection is still a significant clinical challenge. The lack of a gold standard TB diagnostic test has hampered efforts to identify novel biomarkers for use in confirming false-negative IGRA results. Here, results based on screening cohort blood plasma levels of a set of cytokines and chemokines demonstrated that MTB antigen-specific CXCL9 assays could be used to compensate for low IGRA diagnostic accuracy, which occurs when IGRA results obtained for active TB cases are negative; meanwhile, sensitivities of both assays were comparable when used to diagnose active TB patients with positive IGRA results. Subsequent testing of validation cohort samples revealed a CXCL9-based assay sensitivity rate of 95.9%, which was significantly higher than the IGRA sensitivity rate of 74.1%. As consistent with these results, recent results reported by Uzorka and colleagues indicated that approximately two-thirds of patients with borderline QuantiFERON-TB Gold Plus results tested positive for active TB infection using an independent assay based on CXCL9/CXCL10 biomarkers (21), thus indicating that CXCL9 level correlates with MTB infection status.

Despite the fact that negative IGRA results fell below the assay cut-off level, the median IFN-γ level differed only slightly between TB patients with negative IGRAs and healthy controls; this result indicated that TB-specific memory T cell populations were present in IGRA-negative TB patient blood that did not effectively control MTB infection, as reflected by decreased IFN-γ secretion by T cells of these patients. Consequently, there is a critical need to identify new biomarkers that respond more dramatically than IFN-γ to MTB antigen-specific stimulation. For example, CXCL9 is mainly secreted by monocytes in response to IFN-γ stimulation (22), a process that leads to elevated IFN-γ production by T cells of IGRA-negative TB patients that may lead to T cell exhaustion, a phenomenon observed in patients suffering from several types of chronic viral infections (23, 24). Interestingly, T cell exhaustion may support a critical level of pathogen control without causing significant tissue damage. However, prolonged time to culture conversion observed for IGRA-negative patients (as previously reported by our group) suggests that T cell exhaustion occurring in TB patients leads to reduced control over MTB infection *in vivo* (14) that, in turn, indicates that recruitment of immune cells by CXCL9 to infection sites cannot overcome deficient IFN-γ-induced activation of macrophages to control the infection. Nonetheless, additional studies are needed to elucidate underlying mechanisms of T cell exhaustion in IGRA-negative TB patients toward developing effective immunotherapeutic treatments for TB.

As has been reported for CXCL9, CXCL10 is an IFN-γ-induced chemokine that provides promising diagnostic accuracy when used to assess TB status of individuals with false-negative IGRA results. In fact, both CXCL9 and CXCL10 chemokines participate in the host defense against MTB infection, such that their absence has been reported to lead to increased bacillary burden (25). Moreover, results of the current study and results of a previously reported longitudinal study both suggest that the MTB antigen-induced CXCL9 level correlates with active TB disease severity (26), thus implying that CXCL9 and CXCL10 chemokines have important roles in host TB control. Meanwhile, numerous *in vitro* assays currently under development are based on CXCL10 as a biomarker that, in our opinion, may have comparable or lower diagnostic sensitivity in detecting MTB infection than assays based on CXCL9. On the one hand, our results indicated that the CXCL9-based assay detection of MTB-infected subjects was superior to that of the CXCL10-based assay, especially when used to test IGRA-negative TB patients. On the other hand, secretion of CXCL10 is induced by multiple cytokines in addition to IFN-γ, while secretion of CXCL9 exclusively depends on IFN-γ (27) as an explanation for why CXCL9-based assay sensitivity is lower than CXCL10-based assay sensitivity for testing of subjects with impaired IFN-γ secretion. Nonetheless, high CXCL9 assay specificity for discriminating between MTB-infected and uninfected individuals should encourage manufacturers to develop CXCL9-based assays in the future.

This study had several limitations. The first limitation was its small sample size, which was mainly influenced by the limited number of healthy volunteers, which may have weakened the significance of our conclusion. Second, multiple populations at high risk for false-negative IGRA results were not represented in our study cohort, including HIV-infected TB patients, young children, and patients undergoing glucocorticoid therapy. Thus, further validation of the diagnostic accuracy of the CXCL9-based assay should include testing of immunocompromised populations. Third, results of a previous study revealed that the circulating level of CXCL9 differed between patients with pulmonary and extrapulmonary TB disease, thus raising a concern that the TB diagnostic performance of the CXCL9 assay may be unacceptably low when used to test extrapulmonary TB patients. Fourth, despite inclusion of healthy controls with negative IGRA results and no known histories of exposure to tubercle bacilli, elevated antigen-specific CXCL9 levels were noted in a few HC group members that may have resulted from undetected MTB infection or false-positive CXCL9 assay results. Therefore, continuous follow-up monitoring of these individuals should be conducted to better estimate the assay’s positive TB predictive value when used to test members of the general population. Nevertheless, results of the current study highlight the fact that the global TB infection burden has been underestimated due to low IGRA sensitivity as an issue that is preventing effective implementation of TB control measures.

To conclude, our results revealed that use of the MTB antigen-specific CXCL9-based assay described here may compensate for low IGRA diagnostic accuracy when used to test IGRA-negative active TB patients. The sensitivity rate of the CXCL9-based assay (95.9%) was greater than that of the IGRA (74.1%) when used to detect MTB-infected individuals of the validation cohort, thus demonstrating that the antigen-specific CXCL9 assay is a promising alternative to IGRA for use in detecting MTB infection. However, further validation of the diagnostic accuracy of the assay is urgently needed and should be conducted using a larger cohort of patients that includes additional patient populations, such as immunocompromised patients.

## Data availability statement

The raw data supporting the conclusions of this article will be made available by the authors, without undue reservation.

## Ethics statement

This study was approved by the Ethics committee of Beijing Chest Hospital, Capital Medical University (approval number: YJS-2019-016). The guidelines outlined in the Declaration of Helsinki were followed. The studies were conducted in accordance with the local legislation and institutional requirements. The participants provided their written informed consent to participate in this study.

## Author contributions

WR: Writing – original draft, Writing – review & editing. ZM: Data curation, Writing – original draft, Writing – review & editing. QL: Conceptualization, Investigation, Project administration, Software, Validation, Writing – original draft, Writing – review & editing. RL: Methodology, Writing – original draft, Writing – review & editing. LM: Conceptualization, Data curation, Writing – review & editing. CY: Investigation, Writing – original draft, Writing – review & editing. YS: Investigation, Methodology, Writing – original draft, Writing – review & editing. XZ: Methodology, Project administration, Writing – original draft, Writing – review & editing. MG: Investigation, Project administration, Visualization, Writing – original draft, Writing – review & editing. SL: Data curation, Writing – original draft, Writing – review & editing. YP: Writing – original draft, Writing – review & editing.
